# Hand Book of Systematic Urinary Analysis, Chemical and Microscopical

**Published:** 1881-02

**Authors:** 


HAND BOOK OF SYSTEMATIC URINARY ANALYSIS, CHEMICAL AND MICROSCOPICAL. For the use of Physicians, Medical Students and Clinical Assistants. By Frank M. Deems, M.D., Laboratory Instructor in the Medical Department of the University of New York, etc., etc. Industrial Publishing Co., New York. 1880.

This little work is a complete and concise hand book on the subject of urinary analysis, and will be found much more convenient than an unabridged and compendious volume could be for every day analysis. It is gotten up in a convenient semi-pamphlet form, and altogether is a very handy little book.

